# Serum amyloid A circulating levels and disease activity in patients with juvenile idiopathic arthritis

**DOI:** 10.1186/1546-0096-9-S1-P315

**Published:** 2011-09-14

**Authors:** L Cantarini, MG Brizi, G Simonini, T Giani, A Vitale, A Fioravanti, MR Bacarelli, Galeazzi Mauro, R Cimaz

**Affiliations:** 1 Rheumatology Unit, Department of Clinical Medicine and Immunologic Sciences, University of Siena, Italy; 2 Department of Paediatrics, Rheumatology Unit, Anna Meyer Children's Hospital and University of Florence, Italy

## Background

Serum amyloid A (SAA) has been shown to correlate with disease activity in ankylosing spondylitis, and its superiority to erythrocyte sedimentation rate (ESR) and C-reactive protein (CRP) has been suggested.

## Objective

Our aim was to evaluate the association SAA circulating levels and disease activity in patients with juvenile idiopathic arthritis (JIA)

## Methods

Our study group included 41 JIA patients (9 male, 32 female), classified according to the ILAR criteria; 16 had polyarticular disease and 25 had oligoarticular disease; 3/25 patients with oligoarticular diseases had extended oligoarthritis. Serum SAA, ESR and CRP were measured both in patients and 26 healthy controls.

## Results

SAA levels were higher in JIA patients versus healthy controls (p<0.001). Significant positive correlations were found between SAA and the presence of active joints (p<0.05), the number of active joints (p<0.05), ESR (p<0.05) and CRP (p<0.05). No significant correlations between ESR and the presence of active joints (p=0.225) or between ESR and the number of active joints (p=0.520) were demonstrated in JIA patients. No significant correlations were obtained between CRP and the presence of active joints (p=0.855), or between CRP and the number of active joints (p=0.859).

## Conclusion

We found a significant increase of SAA levels in JIA patients as compared to controls, and a strong positive correlation between SAA level and JIA disease activity. We also determined SAA to be a more sensitive laboratory marker than ESR or CRP when compared with the presence and with the number of active joints. We suggest that SAA can be used as an additional indicator of disease activity in JIA.

